# Commentary: Cost-Effectiveness of Left Ventricular Assist Devices as Destination Therapy in the United Kingdom

**DOI:** 10.3389/fcvm.2022.916588

**Published:** 2022-07-11

**Authors:** Priyansh Faldu, Kamal Sharma, Shaival Sharma, Smeet Ramani, Nain Dadhania, Ashwati Konat

**Affiliations:** ^1^MBBS Intern, B.J. Medical College and Civil Hospital, Ahmedabad, India; ^2^Department of Cardiology, SAL Hospital, Ahmedabad, India; ^3^Department of Zoology, Biomedical Technology and Human Genetics, Gujarat University, Ahmedabad, India

**Keywords:** left ventricular assist device (LVAD), heart failure, cost-effectiveness, destination therapy (DT), HeartWare (HVAD), HeartMate II

## Introduction

Recently, there has been a considerable rise in the prevalence of heart failure. As a result, the number of end-stage heart failure patients has increased. While heart transplantation is the most optimal therapy available for these patients, the scarcity of donor organs and the lengthening waiting list have drastically curtailed the number of transplants available. These patients may benefit from left ventricular assist device (LVAD) therapy. LVAD is traditionally used to bridge critical patients to transplantation, but there has been growing debate to consider LVAD for lifelong permanent support termed “destination therapy.” Healthcare systems in countries such as the US and some European countries have started to use LVAD for destination therapy. However, the UK lags in this matter. In the UK, LVADs are only being recommended for bridge-to-transplantation. The Cost-effectiveness of LVAD for DT is considered one of the most important reasons for this practice in the UK. Data from previous studies put the incremental cost-effectiveness ratio (ICER) slightly above £50 000/QALY, which is regarded as cut-off point for interventions in ‘end of life' care in the UK healthcare system. However, these studies have used clinical data from an older generation of LVAD, which is not used nowadays in the current clinical scenario. In this issue, Schueler et al. have made efforts to update the clinical data regarding the cost-effectiveness of newer generation LVADs and have thus, re-open the debate on whether to consider LVAD as a viable option for DT.

## History

Since the REMATCH trial, it has long been established that LVAD therapy is superior to optimal medical management in NYHA class IV HF patients (1). The trial used Thoratec HeartMate at the time, over which several improvements have been made to improve reliability and reduce complications (2). It has been succeeded by HeartMate II and then further by HeartWare. Studies from other European countries estimated ICERs above €80 000, but they also used data from an older generation LVAD, not currently used in the clinical setting (3, 4). Pulikottil-Jacob R et al. (5) compared the cost-effectiveness of HeartWare to HeartMate II, and in their study, HeartWare showed better results compared to HeartMate II. Furthermore, a recent study in the US (6) has also demonstrated improved outcomes compared to previous studies concerning the cost-effectiveness of using LVAD. It is, thus, essential to reevaluate the current cost-effectiveness of HeartWare for DT from the NHS perspective.

## Discussion

Schueler and colleagues have attempted to calculate the present cost-effectiveness of current generation LVAD according to the perspective of NHS payer. They have used a Markov model in end-stage transplant-ineligible patients and have compared the associated costs and QALYs of patients implanted with the HeartWare HVAD System to patients on optimal medical management. Their analysis determined the ICER of around £46 000 per QALY, which makes it cost-effective for the NHS as it is under £50 000, which is the threshold for willingness to pay. Although the model used in the study is routinely practiced, due to the lack of figures from a randomized controlled trial, the authors considered inputs from various other sources and then extrapolated them to match the conditions in the UK.

For the survival data of the MM arm, data from Seattle Heart Failure Model was used. Moreover, for the LVAD cohort, due to the lack of UK LVAD-DT data, it was derived from ENDURANCE Supplemental Trial. But the trial is based on clinical outcomes in the US. So, the outcomes from the trial were then modified to assess the quality of life in the UK. While the data used in the model was contemporary, costs may change from country to country in different healthcare systems. For instance, studies in the US have estimated higher implantation costs and ICER values (7–9). Despite these limitations, all side effects and complications associated with LVAD and their relevant costs in the NHS were considered. Furthermore, in sensitivity analysis, the model results were found to be solid, making the study more credible.

## Conclusion

There have been many advancements in the treatment options for HF patients. While the LVAD therapy has improved patient outcomes, recent modifications have also helped improve the financial aspect of the therapy. The study by Schueler and colleagues has addressed the cost-effectiveness of the latest generation LVAD HeartWare and has shown that LVADs can now be considered for DT even after considering the threshold established in NHS guidelines. However, further study needs to be done through randomized controlled trials to solidify this claim. Considering the limited availability of donor organs for heart transplants, it would be a massive gain for end-stage patients if LVAD is made accessible for DT based on the current cost-effectiveness data.

## Author Contributions

All authors listed have made a substantial, direct, and intellectual contribution to the work and approved it for publication.

## Conflict of Interest

The authors declare that the research was conducted in the absence of any commercial or financial relationships that could be construed as a potential conflict of interest.

## Publisher's Note

All claims expressed in this article are solely those of the authors and do not necessarily represent those of their affiliated organizations, or those of the publisher, the editors and the reviewers. Any product that may be evaluated in this article, or claim that may be made by its manufacturer, is not guaranteed or endorsed by the publisher.

## References

[1] Rose EA Gelijns AC Moskowitz AJ Heitjan DF Stevenson LW Dembitsky W. Long-term use of a left ventricular assist device for end-stage heart failure. New Engl J Med. (2001) 345:1435–43. 10.1056/NEJMoa012175 11794191

[2] Slaughter MS Rogers JG Milano CA Russell SD Conte JV Feldman D. Advanced heart failure treated with continuous-flow left ventricular assist device. New Engl J Med. (2009) 361:2241–51. 10.1056/NEJMoa0909938 19920051

[3] Neyt M Leroy R Devos C Van Brabandt H. Left ventricular assist devices in the treatment of end-stage heart failure. KCE. (2016) 16:264.24482491

[4] Neyt M Van den Bruel A Smit Y De Jonge N Erasmus M Van Dijk D. Cost-effectiveness of continuous-flow left ventricular assist devices. Int J Technol Assess Health Care. (2013) 29:254–60. 10.1017/S0266462313000238 23763844

[5] Pulikottil-Jacob R Suri G Connock M Kandala NB Sutcliffe P Maheswaran H. Comparative cost-effectiveness of the HeartWare versus HeartMate II left ventricular assist devices used in the United Kingdom National Health Service bridge-to-transplant program for patients with heart failure. J Heart Lung Transplant. (2014) 33:350–8. 10.1016/j.healun.2014.01.003 24582838

[6] Silvestry SC Mahr C Slaughter MS Levy WC Cheng RK May DM. Cost-effectiveness of a small intrapericardial centrifugal left ventricular assist device. Asaio J. (2020) 66:862. 10.1097/MAT.0000000000001211 32740129 PMC7386874

[7] Long EF Swain GW Mangi AA. Comparative survival and cost-effectiveness of advanced therapies for end-stage heart failure. Circulat Heart Fail. (2014) 7:470–8. 10.1161/CIRCHEARTFAILURE.113.000807 24563450

[8] Baras Shreibati J Goldhaber-Fiebert JD Banerjee D Owens DK Hlatky MA. Cost-effectiveness of left ventricular assist devices in ambulatory patients with advanced heart failure. JACC: Heart Fail. (2017) 5:110–9. 10.1016/j.jchf.2016.09.008 28017351

[9] Rogers JG Bostic RR Tong KB Adamson R Russo M Slaughter MS. Cost-effectiveness analysis of continuous-flow left ventricular assist devices as destination therapy. Circulat Heart Fail. (2012) 5:10–6. 10.1161/CIRCHEARTFAILURE.111.962951 22052901

